# CODA-ML: context-specific biological knowledge representation for systemic physiology analysis

**DOI:** 10.1186/s12859-019-2812-7

**Published:** 2019-05-29

**Authors:** Mijin Kwon, Soorin Yim, Gwangmin Kim, Saehwan Lee, Chungsun Jeong, Doheon Lee

**Affiliations:** 1 Department of Bio and Brain Engineering, KAIST, 291 Daehak-ro, Yuseong-gu, Daejeon, 34141 Republic of Korea; 2 Bio-Synergy Research Center, 291 Daehak-ro, Yuseong-gu, Daejeon, 34141 Republic of Korea

**Keywords:** Biological knowledge, Essential biological information, Molecular specification, Biological context, Standard language

## Abstract

**Background:**

Computational analysis of complex diseases involving multiple organs requires the integration of multiple different models into a unified model. Different models are often constructed in heterogeneous formats. Thus, the integration of the models requires a standard language format that can effectively represent essential biological information. However, the previously introduced formats have limitations that prevent from adequately representing essential biological information, particularly specifications of bio-molecules and biological contexts.

**Results:**

We defined an XML-based markup language called context-oriented directed association markup language (CODA-ML), which better represents essential biological information. The CODA-ML has two major strengths in designating molecular specifications and biological contexts. It can cover heterogeneous entity types involved in biological events (e.g. gene/protein, compound, cellular function, disease). Molecular types of entities can have molecular specifications which include detailed information of a molecule from isoforms to modifications, enabling high-resolution representation of molecules. In addition, it can distinguish biological events that vary depending on different biological contexts such as cell types or disease conditions. Especially representation of inter-cellular events as well as intra-cellular events is available. These two major strengths can resolve contradictory associations when different models are integrated into one unified model, which improves the accuracy of the model.

**Conclusions:**

With the CODA-ML, diverse models such as signaling pathways, metabolic pathways, and gene regulatory pathways can be represented in a unified language format. Heterogeneous entity types can be covered by the CODA-ML, thus it enables detailed description for the mechanisms of diseases or drugs from multiple perspectives (e.g., molecule, function or disease). The CODA-ML is expected to help integrate different models into one systemic model in an efficient and effective. The unified model can be used to perform computational analysis not only for cancer but also for other complex diseases involving multiple organs beyond a single cell.

**Electronic supplementary material:**

The online version of this article (10.1186/s12859-019-2812-7) contains supplementary material, which is available to authorized users.

## Background

The development of high-throughput experiments such as yeast two-hybrid has led to the discovery of various biological relations that comprise complex biological systems. Systemic analysis of such complex biological systems requires computational modeling that accurately depicts the systems. Especially, complex diseases such as type 2 diabetes involve interactions between multiple organs, tissues, or cells. Therefore, computational analysis for those complex diseases requires the integration of several different models into one unified model to carry out a systemic analysis of multiple organs. To this end, an integrative model needs to cover multiple interconnected organs to enable a comprehensive analysis of the mechanisms of complex diseases or drugs across the whole human body. Moreover, if an integrative model covers heterogeneous biological events between different types of entities such as molecules, cellular functions, or diseases, it would be better for elucidating the mechanisms of diseases or drugs from the perspective of how molecular changes lead to functional changes and then to phenotypic changes.

Different models have been often created in heterogeneous formats, limiting the integration and interoperation among them. To resolve such a problem, it needs a standard format to represent many biological events from different models. The aim of a standard format is to represent the essential information of biological events in an effective and efficient way, which improves the interoperability and interchangeability. To this end, the standard format should be able to represent specifications of molecular types of entities such as molecule type, isoform, epigenetic/post-translational modifications so that an integrated model can capture the details of molecular interactions. In addition, because biological events are often differentially observed depending on biological contexts such as particular organs/tissues/cells or disease conditions, the standard format is expected to distinguish biological events that vary depending on the biological contexts such as anatomical contexts and environmental contexts.

Of the molecular specifications and biological contexts required for a standard format, the former is expected to cover isoforms and modifications of molecular entities. A gene is one of the complicated molecule types with diverse isoforms and modifications. Most of the previous models have not distinguished genes, transcripts, and proteins and have used symbols or ids of their coding genes [1]. Due to the insufficient information for the molecules involved in biological events, computational modeling has faced difficulties in distinguishing genes, transcripts, or proteins. However, state-of-the-art models provide biological events between molecules with their specific types and isoforms. With advancements in sequencing technology, the group for the Genotype-Tissue Expression (GTEx) project recently publicly released co-expression data of gene-gene, gene-transcript, and transcript-transcript pairs across 16 anatomical contexts with the aim to reveal the tissue-specific regulation of transcription and splicing [2]. For example, in the breast mammary, PCOLCE2 gene and a GSN-encoded transcript have functional interactions with a co-expression correlation coefficient of 0.1 [3]. Because detailed information about biological events between specific types of molecules becomes available, it is now possible to differentiate molecules with different isoforms and to create more detailed integrative models.

Molecules can have diverse modifications. Representative modifications of genes, transcripts or proteins include epigenetic/post-translational modifications such as phosphorylation or methylation. These modifications often affect physical interactions with other molecules. For example, ATM-encoded protein increases the amount of BRCA1-encoded protein with a phosphoryl group [4]. Because a protein differently exerts cellular functions depending on the presence of modifications such as phosphorylation, phosphorylated BRCA1-encoded protein should be distinguished from non-phosphorylated BRCA1-encoded protein.

In addition to molecular specifications, biological contexts are also important information to be considered when integrating numerous biological events. Essential groups of biological contexts include anatomical contexts and environmental contexts. First, anatomical contexts refer to organs, tissues or cells at which biological events are observed. Some biological events are generally observed at the majority of anatomical contexts, whereas some others are observed in specific anatomical contexts. Thus, biological events need to be distinguished according to their anatomical contexts. As an example of molecule-function relationships, LEF1 in the hypothalamus is closely associated with hypothalamus development, whereas LEF1 in the blood vessel is rather closely associated with angiogenesis [5]. Anatomical contexts are organized in a hierarchy in which organs are comprised of tissues which are comprised of cells [6]. Therefore, anatomical contexts for a biological event need to be designated in the format of organ, tissue, and cell. A biological event might be differently observed at different cell types in the same organ/tissue or at the same cell type in different organs/tissues. For example, the physical interaction between TSPAN6 and CLEC5A is observed at the glandular cells of the breast whereas it would not be observed at the myoepithelial cells or adipocytes of the breast [1]. Furthermore, a biological event can be a relationship either within a cell or between cells, tissue, or organs [7]. For an example of inter-organ relationships, epinephrine induced in the adrenal glands binds to ADRA2A in the cerebellar cortex. Therefore, standard formats should be able to designate anatomical contexts respectively for each of epinephrine and ADRA2A, enabling to represent relationships between cells, tissue, or organs as well as relationships within a cell.

Environmental contexts refer to conditions such as disease status or medicine treatments under which biological events are observed. Biological events vary depending on the environmental contexts. For example, ESR1 is observed to significantly increase the expression of CCND1 under a breast cancer condition but not in a normal condition [8]. In addition, environmental contexts can be complicatedly combined at the same time, resulting in changes in the relationship types of biological events. In other words, a biological event between two proteins in a certain disease condition might be different from that in normal condition, and it might also be different from that under a combinatorial condition in which a drug is treated under a disease condition [9]. Thus, standard formats need to be able to represent biological events with their single or multiple environmental contexts.

Many groups have put forth efforts to define standard formats for improving interoperation and exchangeability between different models. The most representative formats are the Systems Biology Markup Language (SBML) [10], Biological Expression Language (BEL; http://openbel.org/), and Biological Pathways eXchange (BioPAX) [11]. Even though these formats are widely used, they have limited ability for representing essential biological information, particularly molecular specifications and biological contexts (Table 1). Some previous formats lack room for designating the specifications of biological entities such as isoforms and modifications. For instance, SBML does not provide a way to specify isoforms and modifications. Even though BEL can represent modifications, they do not explicitly state how to differentiate the isoforms. Another previous format, Bio-Synergy Modeling Language (BSML) cannot represent isoforms and modifications [12]. In addition, most of the previous formats have limited ability to represent biological contexts, including both anatomical and environmental contexts. Even though SBML, BEL, and BioPAX are able to represent anatomical contexts, they allow only one anatomical context per biological entity. Thus, the hierarchical organization of the anatomical contexts cannot be represented with these languages (e.g., ‘a protein in cell A of tissue B’). Moreover, none of the three languages consider environmental contexts. Even though they allow user-defined annotations to provide room for non-considered information for each biological relation, user-defined annotations are not in a standard format, limiting the interchangeability of the context information. The absence of a standard format to represent essential biological information, not only biological contexts but also molecular specifications, has limited the exchangeability and interoperation of highly detailed models.

**Table 1:** Comparison with previous standard languages

	SBML	BEL	BioPax	BSML	CODA-ML
Isoform	X	△^a^	O	X	O
Modifications	X	O	O	X	O
Anatomical context	△^b^	△^b^	△^b^	O	O
Environmental context	X	X	X	O	O
Inter-organ/tissue/cell relation	O	O	O	O	O

^a^It does not explicitly mention how to deal with transcript isoforms

^b^It does not represent anatomical contexts in a hierarchical organization, thus only one anatomical context is possible

In this study, we aim to define an XML-based language format, the context-oriented directed association markup language (CODA-ML) that enable precise representation of biological events and effective integrations of different models. Basically, the CODA-ML consists of a triple (i.e. subject, predicate, object) and additional information where the format of a triplet is defined in the Resource Description Framework (RDF - http://www.w3.org/RDF/). Our main goal is to improve the representation of essential biological information, especially molecular specifications and biological contexts. To achieve better representation of molecular specifications, the CODA-ML specifically designate molecule types, isoforms, and modifications of molecules. In addition, the CODA-ML specifies anatomical contexts in a hierarchical organization for each of subject and object in a triple so that inter-cellular events as well as intra-cellular events can be precisely represented. The additional information of a triple includes following components: environmental contexts, the original predicate term in a reference model, species, reference model from which the event comes, and evidence score which is a supportive value for the event. It is expected to create a well-unified large-scale model by integrating biological events from multiple different models in the format of CODA-ML. Some example models are introduced to guide usage of this newly proposed standard language. The unified model can be used for a systemic analysis of mechanisms of drugs or diseases that are involved in multiple organs.

## Method

**Fig. 1:**
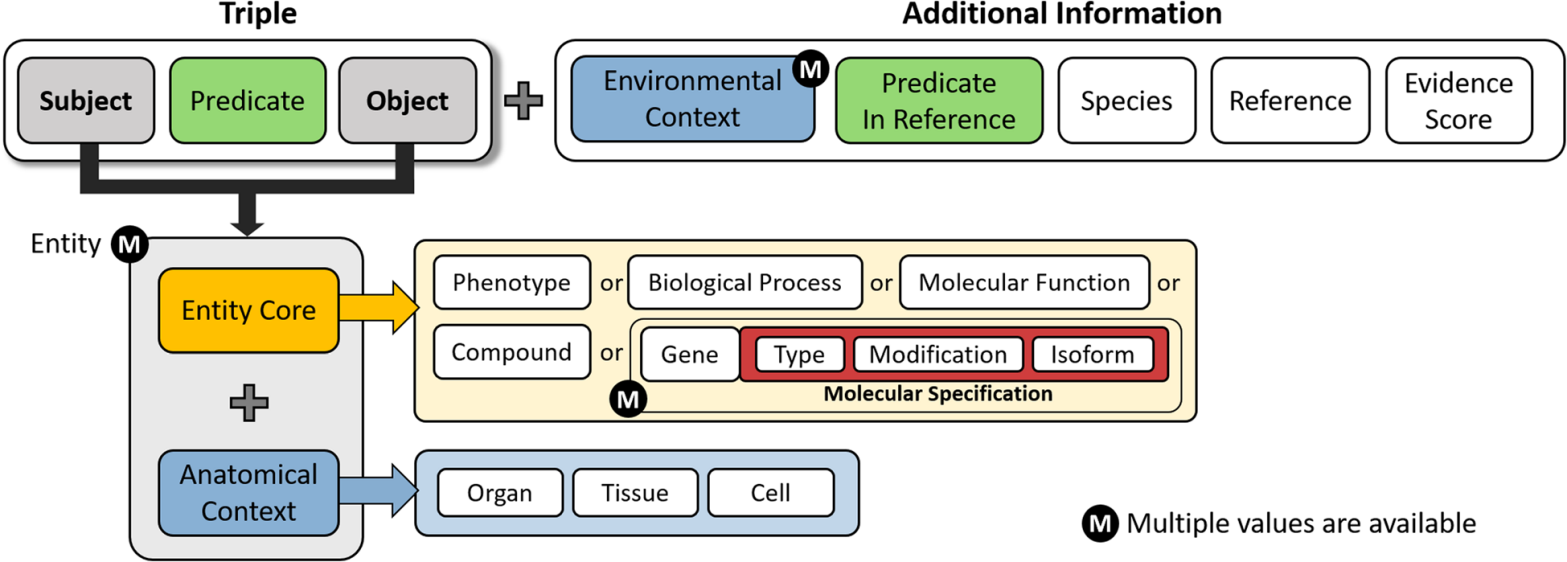
Overview of the CODA-ML. This figure shows overall components comprising the CODA-ML. A knowledge unit (KU) in the CODA-ML format is represented as a triple and its additional information. The triple consists of subject, predicate, and object where each of subject and object has one or more entities. An entity has entity cores and anatomical contexts. The possible entity core types are phenotype, biological process, molecular function, gene, and compound. Gene entity has molecular specifications. The anatomical contexts have an organ, a tissue, and a cell in the form of a hierarchical organization. The additional information of the triple includes environmental contexts, the predicate in reference, species, references, and evidence score

Because each biological event is an atomic unit of the integrative model, it is defined as a knowledge unit (KU). A KU in the CODA-ML format is represented as a triple plus additional information (Fig. 1). A triple consists of a subject, a predicate, and an object in the same way as an RDF. Each subject and object may have one or more entities where an entity consists of entity cores and anatomical contexts. If an entity core is a molecular type, the entity core additionally includes molecular specifications. The predicate can be one of the controlled terms that are pre-defined to represent most of the possible biological relationships. The additional information for a triple includes the environmental contexts, the predicate in the reference, species, reference, and evidence score. A document type definition (DTD) of the CODA-ML describes its structure and ensures a standard way of writing its document (Additional file 1: Figure S1). In the CODA-ML format, biological entities should be written in corresponding identifiers rather than employing plaintexts. The following ontologies are used as reference identifiers: Ensembl [13] for genes, transcripts and proteins, STITCH [14] for compounds, Gene Ontology [15] for biological processes and molecular functions, UMLS [16] for (patho) phenotypes, and Medical Subject Headings (MeSH) [17] for organs, tissues, cells and species. In addition, to illustrate the utility of the CODA-ML, we represent diverse example KUs and example models that elucidate cellular signaling pathways or mechanisms of drug actions.

Detailed elucidation for each component of the CODA-ML format will be in following the order: 1) subject, object, and their molecular specifications, 2) anatomical contexts and environmental contexts, 3) predicate, 4) others (i.e., reference, species, and evidence score). First, each subject and object has one or more entities where an entity consists of entity cores and anatomical contexts. The CODA-ML can cover heterogeneous types of entity cores, including genes, compounds, biological processes, molecular functions, (path) phenotypes (i.e., diseases or symptoms), or other user-defined terms. The entities whose types are genes can additionally have a molecular specification elucidating molecule type, isoform, and modifications. An entity core for a gene, a transcript or a protein is basically represented with an identifier of its coding gene. The three elements of molecular specifications can be filled in according to the available information from the original model (e.g., ‘ENSG00000012048, DNA’ or ‘ENSG00000012048, RNA, ENST00000354071’ where the Ensembl identifiers for ‘BRCA1’ and one of its transcripts are, respectively, ‘ENSG00000012048’ and ‘ENST00000354071’). For an example that given information is insufficient to fill in molecular specification, when a BRCA1-originated protein whose isoform is specified physically interacts with a BAP1-originated protein whose isoform is not specified, the subject and the object of this KU become respectively ‘BRCA1, protein, ENSP00000312236’ and ‘BAP1, protein’ (Additional file 1: Figure S2). DNAs, RNAs, or proteins can have epigenetic or post-translational modifications (e.g., phosphorylation or methylation). Considering such molecular modifications, this format distinguishes molecules with and without modifications (e.g. ‘ENSG00000012048, protein, ENSP00000312236’) ≠ ‘ENSG00000012048, protein, ENSP00000312236, phosphorylated’). Some entities may have multiple modifications at the same time (e.g. ‘ENSG00000 012048, protein, ENSP00000312236, phosphorylated/methylated’).

Each entity has three anatomical contexts in the format of the hierarchical organization: an organ, a tissue, and a cell. In the case of an event observed within an anatomical location (i.e., an intra-cellular event) both of subject and object have identical anatomical contexts. For example, because luteinizing hormone receptor (LHCGR) in the ovary is related to primary follicle growth in the ovary [15], subject and object of this KU identically have the ‘D010053’ as anatomical contexts where the MeSH identifier for the ovary is ‘D010053’ (Additional file 1: Figure S3). On the other hand, in the case that an event is an interaction between entities in different anatomical contexts (i.e., an inter-cellular event), subject and object of this KU have different anatomical contexts. For example, because luteinizing hormone produced in the pituitary gland induces estrogen in the granulosa cell of the ovary [7], anatomical contexts for the subject and the object of this KU, respectively, are ‘D010902’ (pituitary gland) and ‘D010053, D006107’ (ovary, granulosa cell) (Additional file 1: Figure S4). Inter-cellular events bridge not only cells but also tissues and organs that contain them. A KU can be represented without anatomical contexts in the CODA-ML if the modeling purposes do not require anatomical contexts specificity or if the original resources do not designate sufficient anatomical information for an event (e.g., the protein ESR1 physically interacts with the protein BRCA1 [18] (Additional file 1: Figure S5), or ESR1 is a marker or mechanism of breast cancer [19] (Additional file 1: Figure S6)).

Whereas anatomical contexts are specified for each of subject and object, environmental contexts are specified for a triple (subject, predicate, and object). A KU with no environmental contexts implies that this biological event is observed in a normal condition without any perturbations. Each KU may have zero or more environmental contexts such as disease condition, cell line condition, drug treatment, siRNA treatment, or herb treatment. For example, because the protein ESR1 increases the expression level of CCND1 in a breast cancer condition [4], the environmental context for this KU becomes ‘phenotype’-‘C0678222’ where the MeSH id for ‘breast cancer’ is ‘C0678222’ (Additional file 1: Figure S7). As another example, because resveratrol was confirmed to increase the cellular accumulation of doxorubicin in the MCF-7 cell line [20], the environmental context for this KU becomes ‘cell line’-‘CLO0007606’ where the CLO id for ‘MCF-7’ is ‘CLO0007606 ‘(Additional file 1: Figure S8).

A typical entity consists of a single entity core and the anatomical contexts, but some entities may involve multiple entity cores. For example, because a protein complex composed of CCND1 (ENSG00000110092) and CDK4 (ENSG00000135446) phosphorylates RB1-encoded protein [4], subject of this KU has two entity cores, ‘ENSG00000110092, protein’ and ‘ENSG00000135446, protein’ (Additional file 1: Figure S9). Furthermore, the representative cases that require multiple entities for a subject and an object are metabolic reactions. For example, the testicular 17-beta-hydroxysteroid dehydrogenase is defined with ‘Estrone + H + NADPH <=> Estradiol + NADP’ [21]. Here, the subject of this KU becomes ‘CIDs00005870’, ‘CIDs00001038’ and ‘CIDs22833512’ whereas the object becomes ‘CIDs00005757’ and ‘CIDs00005886’ (Additional file 1: Figure S10).

**Fig. 2:**
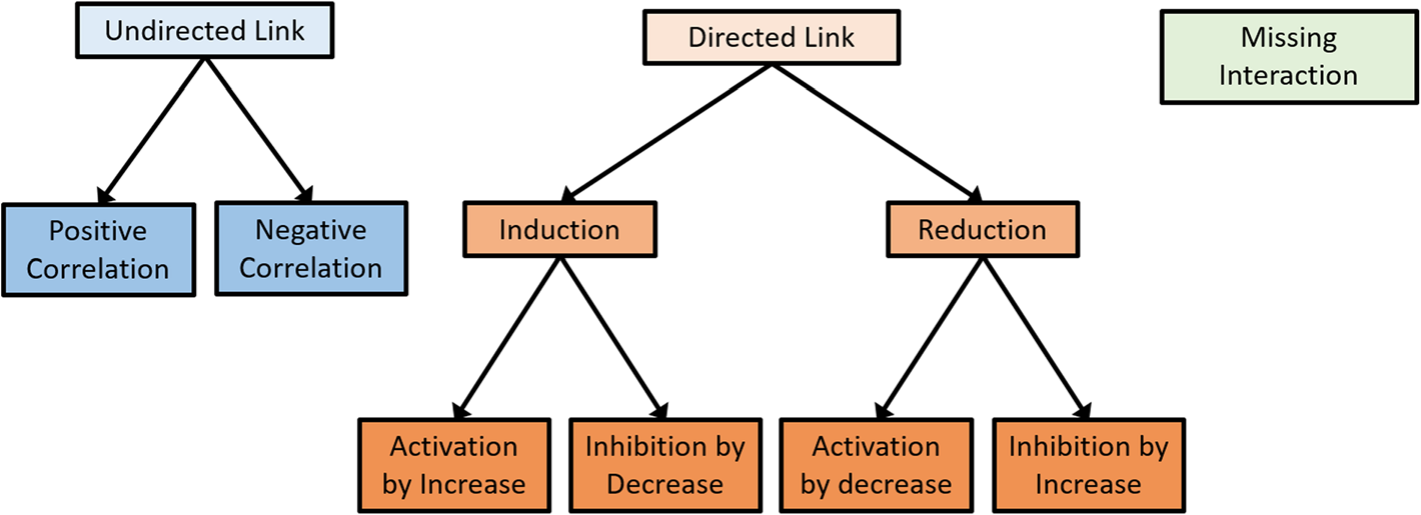
Hierarchical relations between 11 controlled predicate terms. It shows hierarchical relations between 11 exclusive terms that are pre-defined depending on the resolution of direction and sign. Based on the causality of subject and object in a knowledge unit (KU), the predicate of the KU becomes either of ‘undirected link’ or ‘directed link’. Furthermore, if the more detailed information for the sign is available, the KU can have higher-resolution terms such as ‘positive increase’. In such cases that a biological event existing in normal condition is observed to disappear under certain conditions such as a disease, the predicate of this KU can be ‘missing interaction’

Previous models often use non-standardized terms to indicate the relationships of biological events, which may result in several different terms with the same meaning. For example, two differently written terms, ‘up-regulate’ and ‘express’ ultimately mean the same biological relationship. An original term of a predicate from a reference model (e.g., up-regulate or express) is denoted for the ‘predicate in the reference’ in additional information. On the other hand, the predicate in a triple is represented by one of 11 controlled vocabularies that are pre-defined depending on the resolution of the direction and sign: directed link, induction, reduction, activation by increase, activation by decrease, inhibition by increase, inhibition by decrease, undirected link, positive correlation, negative correlation and missing interaction. The relations between the 11 controlled terms are depicted in Fig. 2. Lower-level terms (e.g., activation/inhibition by increase/decrease, positive/negative correlation) provide higher-resolution information than the other higher-level terms (e.g., directed link, undirected link). Unlike ‘undirected link’, the ‘directed link’ refers to a relationship with known causality from the subject to the object. The ‘activation/inhibition by increase/decrease’ represents a relationship in which the increased/decreased regulation or activity of subject results in the activated/inhibited regulation or activity of object. The ‘directed link’ can be used for the predicate of a KU whose causality is known, but the detailed sign is not confirmed. In such cases that a biological event existing in a normal condition is observed to disappear under a certain environmental context such as a disease condition, predicate of this KU can be a ‘missing interaction’. The ‘predicate in the reference’ for ‘activation by increase’ possibly would have the following words: ‘activate’, ‘express’, ‘positively regulate’ or ‘increase’.

The additional information of a triple in a KU includes the following components: environmental contexts (explained above), the predicate in the reference (explained above), species, reference, and evidence score. First, each KU has a species which the corresponding biological events are observed from (e.g., *Homo sapiens* or mouse). Second, the ‘reference’ describes detailed information of the reference models which the KUs come from. Here, a reference consists of the following multiple elements: reference type, name, description, record id, version, and acquisition date. The ‘reference type’ indicates a type of a reference model (e.g., database, literature, or wet experiment). The ‘name’ refers to the exact name of a reference model (e.g., BioGrid, KEGG pathway, PubMed, or in vitro). The ‘description’ contains a plaintext explanation for the reference model (e.g., ‘a manually curated database’ or ‘yeast-two hybrid’) or the corresponding sentence of the referred article in the case of a literature type. The ‘record id’ refers to an URL link that detailed information of the KU is available (e.g. ‘https://www.genome.jp/dbget-bin/www_bget?hsa00010’ for a KU from ‘KEGG pathway’ model). The ‘version’ refers to the version or updated date of the databases, the published date of the literature, or an experiment-carried out date. Lastly, the ‘acquisition date’ refers to a date when a biological event from a reference model is converted into KU in the CODA-ML format. For example, a KU is defined based on a biological event from the hsa05224 of the KEGG pathway [4], which is, a database containing manually curated data that was lastly updated in 2017. Its reference has the following elements and values: ‘reference type’-‘database’, ‘name’-‘KEGG’, ‘description’-‘manually curated data’, ‘record id’-‘http://www.genome.jp/kegg-bin/show_pathway?hsa05224’, ‘version’-‘2017’, ‘acquisition date’-‘2018’ (Additional file 1: Figure S9). Last, to support the reliability, each KU can have an ‘evidence score’ which is either a discrete or continuous value based on given information from the reference model (e.g., *p*-value).

## Results

The CODA-ML was defined to precisely represent essential biological information of biological events, especially molecular specifications and biological contexts whose representation was limited by other language formats. For molecular specifications, molecule type, isoform, and modifications are included. For biological contexts, anatomical contexts and environmental contexts are covered. Thus, the CODA-ML can distinguish different isoforms and molecules with or without modifications, differentiate biological events under different anatomical or environmental context, and cover inter-cellular events as well as intra-cellular events (Table 1).

**Fig. 3:**
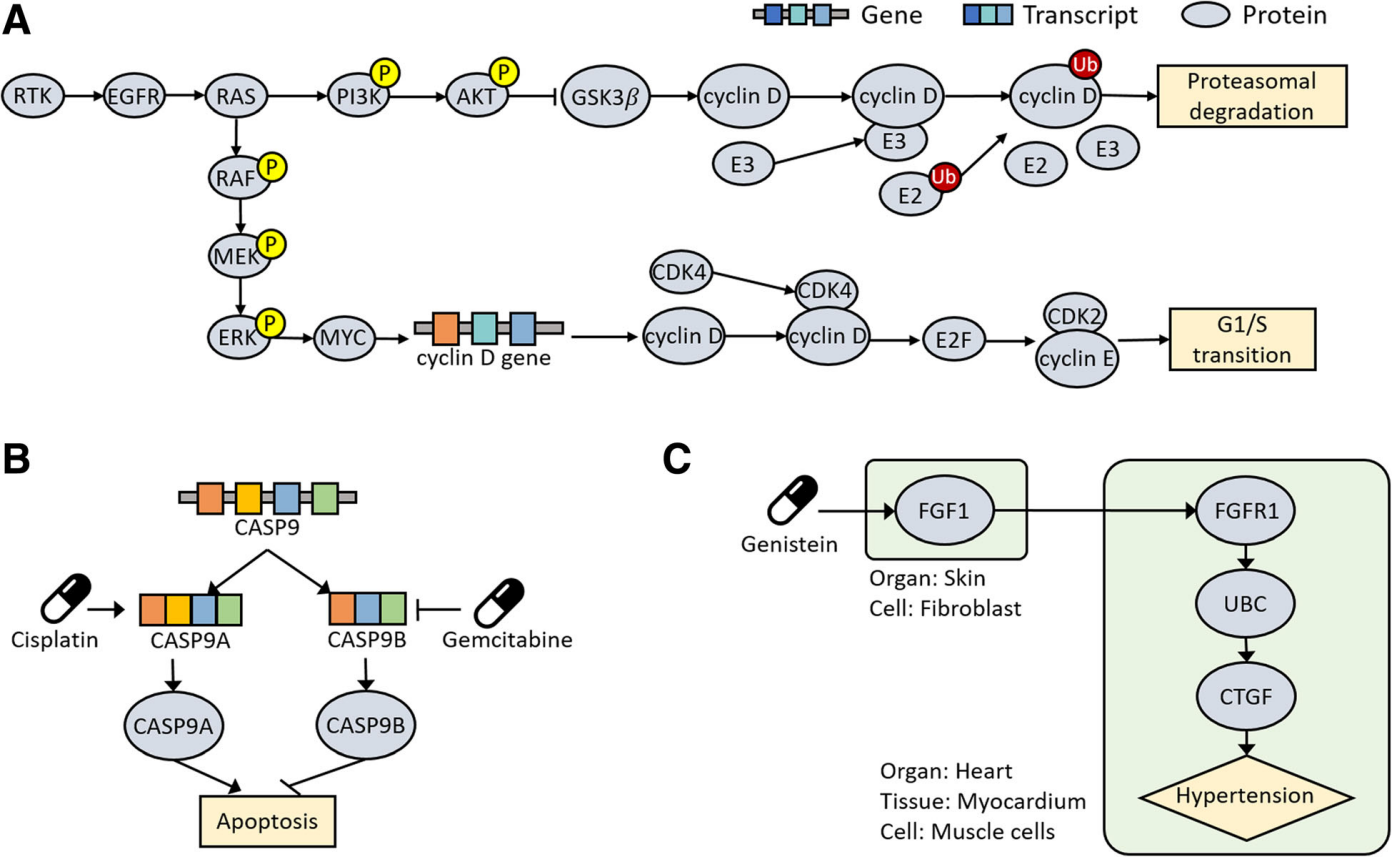
Three example models in the CODA-ML format. To evaluate the utility of the CODA-ML format, three different examples are introduced. **a** The RTK signaling pathway includes the MAPK / ERK and PI3K / AKT signaling pathways linked to two cancer-related functions. Many molecules in this pathway have molecular modifications or are protein complexes, which requires molecular representation in detail. **b** Different isoforms originated from the same gene may have different effects to the same function. The mechanisms of the actions of cisplatin and gemcitabine show that drugs may target different isoforms, which highlights that molecular isoforms are essential for precise representation of mechanisms of drug actions. **c** The mechanism of genistein actions for hypertension was inferred by a previous model in a computational way. This mechanism shows that mechanisms of drug actions particularly, for complex diseases, may involve inter-anatomical relationships as well as intra-anatomy relationships

To evaluate the utility of the CODA-ML format and make a user guide for this, three different examples are introduced. The first example is part of the RTK signaling pathway, involving the MAPK/ERK and PI3K/AKT pathways, which induce two cancer-related cell functions; proteasomal degradation and G1/S transition [22]. The RTK signaling pathways include diverse kinases such as MEK and ERK that phosphorylate other proteins. Thus, molecular specification, particularly molecular modifications, are important to precisely represent this pathway. Illustrations of this pathway considering molecular specifications are depicted in Fig. 3a.

The second example is the mechanism of the actions of cisplatin and gemcitabine [23]. Two CASP9-encoded protein isoforms, CASP9a and CASP9b, have different effects on apoptosis. CASP9a induces apoptosis whereas CASP9b inhibits this. To promote apoptosis, cisplatin increases the amount of CASP9a, and gemcitabine decreases the activity of CASP9b (Fig. 3b). Because drugs may target different isoforms, mechanisms of drug actions can be elucidated in more detail by using molecular isoforms.

The last example is the mechanism of genistein actions for hypertension inferred by a previous model [24]. According to the inference, genistein binds to the FGF1 protein in fibroblasts of the skin; the FGF1 protein activates the FGFR1 protein in the muscle cells of the heart; FGFR1 protein activates the UBC protein; the UBC protein activates the CTGF protein, and CTGF affects hypertension (Fig. 3c). Because mechanisms of drug actions can be related to inter-cellular biological events, to represent the anatomical contexts for each of subject and object helps to better elucidate drug mechanisms. These three examples models in the CODA-ML format can be accessed via http://github.com/MijinKwon/CODA-ML.

For the interoperability and interchangeability of the CODA-ML with the most representative formats previously introduced (i.e., SBML, BioPax, and BEL), we provide a conversion module. There are modules that convert language formats from BioPax to SBML or BEL and vice versa [21, 25], thus we provide a new module that can convert language formats between CODA-ML and BioPax. The corresponding codes are also available at: http://github.com/MijinKwon/CODA-ML.

## Discussion / conclusion

We defined an XML-based language format called the CODA-ML that effectively represents the essential information of biological events and fosters interoperability and exchangeability among different models with heterogeneous formats. Among the several essential biological information, the major focuses of this work were molecular specifications (i.e. molecule type, isoform, and modifications) and biological contexts (i.e. anatomical contexts and environmental contexts). The two information is very necessary for biological events but previous formats had limited ability to represent them. Using molecular specifications, the CODA-ML discriminates genes, transcripts, and proteins with or without modifications, and thus, it facilitates the detailed elucidation of mechanism pathways. The usefulness of molecular specification was proved by example models of RTK signaling pathway and mechanisms of actions of cisplatin and gemcitabine.

In addition, the CODA-ML designates anatomical contexts for each of subject and object in the format of hierarchical organization. Thus, it can represent inter-anatomical events as well as intra-anatomical events, enabling a comprehensive analysis of the mechanisms of complex diseases or drug actions that are involved in multiple organs. The utility of designating anatomical contexts per entity was proved by mechanisms of action of genistein which is used for hypertension, one of the most representative diseases. Furthermore, consideration of environmental contexts, which have not been appreciated by previously introduced standard formats, enables the distinction of associations that are differently observed depending on the conditions such as disease status or drug treatment. As shown in the three examples, the consideration of molecular specifications and biological contexts could help more precise representation of biological events. These results demonstrate that the CODA-ML outperforms than previous language formats in unifying biological events with detailed biological information. The CODA-ML is expected to be a great help to the effective integration of different individual models and comprehensive analysis of complex diseases.

Although we put forth many efforts to propose a new standard format to effectively represent biological events, there could be some unresolved issues. First, while the CODA-ML can standardize complicated but essential biological information of biological events, some other important information (e.g., gene sequence) was not covered for user convenience and simplicity of the representation, possibly restricting the performance of models for very high-resolution analyses. Second, from an opposite perspective, essential information that the CODA-ML requires may often be unavailable, restricting the utility of this format. Because the CODA-ML is of the XML-based extendable format, it can be freely modified at the user’s discretion if needed.

## Additional file

Additional file 1: Supplementary materials containing one document type definition (DTD) of the CODA-ML (Figure S1) and nine knowledge unit (KU) examples (Figure S2-S10). (DOCX 61 kb)

## Abbreviations

CODA-ML: Context-oriented directed association markup language
KU: Knowledge unit

## References

[1] Chatr-Aryamontri A, Breitkreutz BJ, Oughtred R, Boucher L, Heinicke S, Chen D, Stark C, Breitkreutz A, Kolas N, O'Donnell L (2015). The BioGRID interaction database: 2015 update. Nucleic Acids Res.

[2] Saha A, Kim Y, Gewirtz ADH, Jo B, Gao C, McDowell IC, Consortium GT, Engelhardt BE, Battle A (2017). Co-expression networks reveal the tissue-specific regulation of transcription and splicing. Genome Res.

[3] Zerbino DR, Achuthan P, Akanni W, Amode MR, Barrell D, Bhai J, Billis K, Cummins C, Gall A, Giron CG (2018). Ensembl 2018. Nucleic Acids Res.

[4] Kanehisa M, Goto S (2000). KEGG: Kyoto encyclopedia of genes and genomes. Nucleic Acids Res.

[5] Greene CS, Krishnan A, Wong AK, Ricciotti E, Zelaya RA, Himmelstein DS, Zhang R, Hartmann BM, Zaslavsky E, Sealfon SC (2015). Understanding multicellular function and disease with human tissue-specific networks. Nat Genet.

[6] Hunter PJ, Thomas K (2003). Borg: integration from proteins to organs: the Physiome project. Nat Rev Mol Cell Biol.

[7] Dönitz J, Wingender E (2014). EndoNet: an information resource about the intercellular signaling network. BMC Syst Biol.

[8] Jeon S, Choi JY, Lee KM, Park SK, Yoo KY, Noh DY, Ahn SH, Kang D (2010). Combined genetic effect of CDK7 and ESR1 polymorphisms on breast cancer. Breast Cancer Res Treat.

[9] Hensler JG (2002). Differential regulation of 5-HT1A receptor-G protein interactions in brain following chronic antidepressant administration. Neuropsychopharmacology.

[10] Chaouiya C (2013). SBML qualitative models: a model representation format and infrastructure to foster interactions between qualitative modelling formalisms and tools. BMC Syst Biol.

[11] Demir E, Cary MP, Paley S, Fukuda K, Lemer C, Vastrik I, Wu G, D'Eustachio P, Schaefer C, Luciano J (2010). The BioPAX community standard for pathway data sharing. Nat Biotechnol.

[12] Hwang W (2013). BSML: bio-synergy modeling language for multi-component and multi-target analysis.

[13] Yates A, Akanni W, Amode MR, Barrell D, Billis K, Carvalho-Silva D, Cummins C, Clapham P, Fitzgerald S, Gil L (2015). Ensembl 2016. Nucleic Acids Res.

[14] Szklarczyk D, Santos A, von Mering C, Jensen LJ, Bork P, Kuhn M (2015). STITCH 5: augmenting protein–chemical interaction networks with tissue and affinity data. Nucleic Acids Res.

[15] Harris MA, Clark J, Ireland A, Lomax J, Ashburner M, Foulger R, Eilbeck K, Lewis S, Marshall B, Mungall C (2004). The gene ontology (GO) database and informatics resource. Nucleic Acids Res.

[16] Bodenreider O (2004). The unified medical language system (UMLS): integrating biomedical terminology. Nucleic Acids Res.

[17] Coletti MH, Bleich HL (2001). Medical subject headings used to search the biomedical literature. J Am Med Inform Assoc.

[18] Fan S (2001). Role of direct interaction in BRCA1 inhibition of estrogen receptor activity. Oncogene.

[19] Davis AP, Grondin CJ, Johnson RJ, Sciaky D, King BL, McMorran R, Wiegers J, Wiegers TC, Mattingly CJ (2017). The comparative Toxicogenomics database: update 2017. Nucleic Acids Res.

[20] Kim TH, Shin YJ, Won AJ, Lee BM, Choi WS, Jung JH, Chung HY, Kim HS (2014). Resveratrol enhances chemosensitivity of doxorubicin in multidrug-resistant human breast cancer cells via increased cellular influx of doxorubicin. Biochim Biophys Acta.

[21] Hoyt CT, Konotopez A, Ebeling C (2017). PyBEL: a computational framework for biological expression language. Bioinformatics.

[22] VanArsdale T, Boshoff C, Arndt KT, Abraham RT (2015). Molecular pathways: targeting the cyclin D-CDK4/6 axis for cancer treatment. Clin Cancer Res.

[23] Rehman SU, Husain MA, Sarwar T, Ishqi HM, Tabish M (2015). Modulation of alternative splicing by anticancer drugs. Wiley Interdiscip Rev.

[24] Yu H, Jung J, Yoon S, Kwon M, Bae S, Yim S, Lee J, Kim S, Kang Y, Lee D (2018). Publisher correction: CODA: integrating multi-level context-oriented directed associations for analysis of drug effects. Sci Rep.

[25] Demir E, Babur Ö, Rodchenkov I, Aksoy BA, Fukuda KI, Gross B, Sümer OS, Bader GD, Sander C (2013). Using biological pathway data with paxtools. PLoS Comput Biol.

