# Supplementary material for: CODA-ML: context-specific biological knowledge representation for systemic physiology analysis
Source: BMC Bioinformatics. 2019 May 29;20(Suppl 10):248. doi: 10.1186/s12859-019-2812-7 (PMC6538558; doi:10.1186/s12859-019-2812-7)
Supplement: Supplementary file 1 — Supplementary materials containing one document type definition (DTD) of the CODA-ML (Figure S1) and nine knowledge unit (KU) examples (Figure S2-S10). (DOCX 61 kb) [file 12859_2019_2812_MOESM1_ESM.docx]

**CODA-ML: Context-Specific Biological Knowledge Representation for Systemic Physiology Analysis**

Mijin Kwon^1^, Soorin Yim^1^, Gwangmin Kim^1^, Saehwan Lee^1^, Choongsoon Jeong^1^ and Doheon Lee^1,2,*^

^1^ Department of Bio and Brain Engineering, KAIST, 291 Daehak-ro, Yuseong-gu, Daejeon, 305-701, Republic of Korea ^2^ Bio-Synergy Research Center, 291 Daehak-ro, Yuseong-gu, Daejeon, 305-701, Daejeon, Republic of Korea

***** Corresponding author

| <!ELEMENT CODA_Knowledge_Units (CODA_Knowledge_Unit+)>  <!ELEMENT CODA_Knowledge_Unit (Subject,Predicate,Object,Environmental_Context?,Predicate_In_Reference*,Species?,Reference+,Evidence_Score?)>  <!ELEMENT Subject (Entity+)>  <!ELEMENT Entity (Entity_Core,Anatomical_Context?)>  <!ELEMENT Entity_Core (Phenotype\|Biological_Process\|Molecular_Function\|Compound\|Gene +)>  <!ELEMENT Phenotype (Ref_Identifier+)>  <!ELEMENT Ref_Identifier (DBName, Identifier)>  <!ELEMENT DBName (#PCDATA)>  <!ELEMENT Identifier (#PCDATA)>  <!ELEMENT Biological_Process (Ref_Identifier+)>  <!ELEMENT Molecular_Function (Ref_Identifier+)>  <!ELEMENT Compound (Ref_Identifier+)>  <!ELEMENT Gene (Ref_Identifier+,Molecule_Specification?)>  <!ELEMENT Molecule_Specification (Molecule_Type?,Molecule_Modification*,Molecule_Isoform?)>  <!ELEMENT Molecule_Type (#PCDATA)>  <!ELEMENT Molecule_Modification (#PCDATA)>  <!ELEMENT Molecule_Isoform (Ref_Identifier+)>  <!ELEMENT Anatomical_Context (Organ?,Tissue?,Cell?)>  <!ELEMENT Organ (Ref_Identifier+)>  <!ELEMENT Tissue (Ref_Identifier+)>  <!ELEMENT Cell (Ref_Identifier+)>  <!ELEMENT Predicate (#PCDATA)>  <!ELEMENT Object (Entity+)>  <!ELEMENT Environmental_Context (Condition*)>  <!ELEMENT Condition (Key,Value)>  <!ELEMENT Key (#PCDATA)>  <!ELEMENT Value (Ref_Identifier+)>  <!ELEMENT Predicate_In_Reference (#PCDATA)>  <!ELEMENT Species (Ref_Identifier+)>  <!ELEMENT Reference (Reference_Type?,Name?,Description?,Record_ID?,Version?,Acquisition_Date?)>  <!ELEMENT Reference_Type (#PCDATA)>  <!ELEMENT Description (#PCDATA)>  <!ELEMENT Record_ID (#PCDATA)>  <!ELEMENT Version (#PCDATA)>  <!ELEMENT Acquision_Date (#PCDATA)>  <!ELEMENT Evidence_Score (Item*)>  <!ELEMENT Item (Key, Score)>  <!ELEMENT Score (#PCDATA)> |
| --- |

**Figure S1 A document type definition (DTD) of the CODA-ML.** This figure describes the structure of the language and ensuring a standard way of writing its document. It mainly consists of a triple with additional information. A triple is composed of subject, predicate, and object where each of subject and object has one or more entities. Additional information includes predicate in reference, environmental contexts, species, references, and evidence score.

| <CODA_Knowledge_Unit>  <Subject>  <Entity>  <Entity_Core>  <Gene>  <Ref_Identifier>  <DBName>Ensembl</DBName>  <Identifier>ENSG00000012048</Identifier>  </Ref_Identifier>  <Molecule_Specification>  <Molecule_Type>Protein</Molecule_Type>  <Molecule_Isoform>  <Ref_Identifier>  <DBName>Ensembl</DBName>  <Identifier> ENSP00000312236</Identifier>  </Ref_Identifier>  </Molecule_Isoform>  </Molecule_Specification>  </Gene>  </Entity_Core>  </Entity>  </Subject>  <Predicate>Undirected link</Predicate>  <Object>  <Entity>  <Entity_Core>  <Gene>  <Ref_Identifier>  <DBName>Ensembl</DBName>  <Identifier>ENSG00000163930</Identifier>  </Ref_Identifier>  <Molecule_Specification>  <Molecule_Type>Protein</Molecule_Type>  </Molecule_Specification>  </Gene>  </Entity_Core>  </Entity>  </Object>  <Predicate_In_Reference>Protein-protein interaction</Predicate_In_Reference>  <Species>  <Ref_Identifier>  <DBName>MeSH</DBName>  <Identifier>D006801</Identifier>  </Ref_Identifier>  </Species>  <Reference>  <Reference_Type>Database</Reference_Type>  <Name>neXtProt</Name>  <Description>Manually curated data</Description>  <Record_ID>www.nextprot.org/entry/NX_P38398/interactions?isoform=NX_P38398-5</Record_ID>  <Version>2017</Version>  <Acquisition_Date>2017</Acquisition_Date>  </Reference>  <Evidence_Score>  <Item>  <Key>Manual curation</Key>  <Score>1</Score>  </Item>  </Evidence_Score>  </CODA_Knowledge_Unit> |
| --- |

**Figure S2 Knowledge Unit (KU) Example 1.** One isoform of BRCA1 (ENSG00000012048), referred as ENSP00000312236, physically interacts with the protein encoded by BAP1 (ENSG00000163930).

| <CODA_Knowledge_Unit>  <Subject>  <Entity>  <Entity_Core>  <Gene>  <Ref_Identifier>  <DBName>Ensembl</DBName>  <Identifier>ENSG00000138039</Identifier>  </Ref_Identifier>  </Gene>  </Entity_Core>  <Anatomical_Context>  <Organ>  <Ref_Identifier>  <DBName>MeSH</DBName>  <Identifier>D010053</Identifier>  </Ref_Identifier>  </Organ>  </Anatomical_Context>  </Entity>  </Subject>  <Predicate>Undirected link</Predicate>  <Object>  <Entity>  <Entity_Core>  <Biological_Process>  <Ref_Identifier>  <DBName>GO</DBName>  <Identifier>GO:0001545</Identifier>  </Ref_Identifier>  </Biological_Process>  </Entity_Core>  <Anatomical_Context>  <Organ>  <Ref_Identifier>  <DBName>MeSH</DBName>  <Identifier>D010053</Identifier>  </Ref_Identifier>  </Organ>  </Anatomical_Context>  </Entity>  </Object>  <Predicate_In_Reference>Associate</Predicate_In_Reference>  <Species>  <Ref_Identifier>  <DBName>MeSH</DBName>  <Identifier>D006801</Identifier>  </Ref_Identifier>  </Species>  <Reference>  <Reference_Type>Database</Reference_Type>  <Name>GO</Name>  <Description>Gene association with GO in human</Description>  <Record_ID>amigo.geneontology.org/amigo/term/GO:0001545 </Record_ID>  <Version>2015</Version>  <Acquisition_Date>2017</Acquisition_Date>  </Reference>  <Evidence_Score>  <Item>  <Key>Manual curation</Key>  <Score>1</Score>  </Item>  </Evidence_Score>  </CODA_Knowledge_Unit> |
| --- |

**Figure S3 Knowledge Unit (KU) Example 2.** LHCGR, luteinizing hormone receptor (ENSG00000138039), is related to primary ovarian follicle growth (GO:0001545).

| <CODA_Knowledge_Unit>  <Subject>  <Entity>  <Entity_Core>  <Gene>  <Ref_Identifier>  <DBName>Ensembl</DBName>  <Identifier>ENSG00000104826 </Identifier>  </Ref_Identifier>  <Molecule_Specification>  <Molecule_Type>Protein</Molecule_Type>  <Molecule_Isoform>  <Ref_Identifier>  <DBName>Ensembl</DBName>  <Identifier> ENSP00000221421</Identifier>  </Ref_Identifier>  </Molecule_Isoform>  </Molecule_Specification>  </Gene>  </Entity_Core>  <Anatomical_Context>  <Organ>  <Ref_Identifier>  <DBName>MeSH</DBName>  <Identifier>D010902</Identifier>  </Ref_Identifier>  </Organ>  </Anatomical_Context>  </Entity>  </Subject>  <Predicate>Positive increase</Predicate>  <Object>  <Entity>  <Entity_Core>  <Compound>  <Ref_Identifier>  <DBName>Ensembl</DBName>  <Identifier>CIDs00448537</Identifier>  </Ref_Identifier>  </Compound>  </Entity_Core>  <Anatomical_Context>  <Organ>  <Ref_Identifier>  <DBName>MeSH</DBName>  <Identifier>D010053</Identifier>  </Ref_Identifier>  </Organ>  <Cell>  <Ref_Identifier>  <DBName>MeSH</DBName>  <Identifier>D006107</Identifier>  </Ref_Identifier>  </Cell>  </Anatomical_Context>  </Entity>  </Object>  <Predicate_In_Reference>Associate</Predicate_In_Reference>  <Species>  <Ref_Identifier>  <DBName>MeSH</DBName>  <Identifier>D006801</Identifier>  </Ref_Identifier>  </Species>  <Reference>  <Reference_Type>Database</Reference_Type>  <Name>EndoNet</Name>  <Description>Manually curated data</Description>  <Record_ID>endonet.bioinf.med.uni-goettingen.de/hormone/ENH00038 </Record_ID>  <Version>2017</Version>  <Acquisition_Date>2017</Acquisition_Date>  </Reference>  <Evidence_Score>  <Item>  <Key>Manual curation</Key>  <Score>1</Score>  </Item>  </Evidence_Score>  </CODA_Knowledge_Unit> |
| --- |

**Figure S4 Knowledge Unit (KU) Example 3.** Secretion of luteinizing hormone (ENSP00000221421) from pituitary gland (D010902) results in increase of estrogen (CIDs00448537) in granulosa cells (D006107) in ovary (D010053).

| <CODA_Knowledge_Unit>  <Subject>  <Entity>  <Entity_Core>  <Gene>  <Ref_Identifier>  <DBName>Ensembl</DBName>  <Identifier>ENSG00000091831</Identifier>  </Ref_Identifier>  <Molecule_Specification>  <Molecule_Type>Protein</Molecule_Type>  </Molecule_Specification>  </Gene>  </Entity_Core>  </Entity>  </Subject>  <Predicate>Undirected link</Predicate>  <Object>  <Entity>  <Entity_Core>  <Gene>  <Ref_Identifier>  <DBName>Ensembl</DBName>  <Identifier>ENSG00000012048</Identifier>  </Ref_Identifier>  <Molecule_Specification>  <Molecule_Type>Protein</Molecule_Type>  </Molecule_Specification>  </Gene>  </Entity_Core>  </Entity>  </Object>  <Predicate_In_Reference>Protein-protein interaction</Predicate_In_Reference>  <Species>  <Ref_Identifier>  <DBName>MeSH</DBName>  <Identifier>D006801</Identifier>  </Ref_Identifier>  </Species>  <Reference>  <Reference_Type>Literature</Reference_Type>  <Name>PubMed</Name>  <Description>Data curated from biomedical literature</Description>  <Record_ID>https://www.ncbi.nlm.nih.gov/pubmed/11244506 </Record_ID>  <Version>2001</Version>  <Acquisition_Date>2017</Acquisition_Date>  </Reference>  <Reference>  <Reference_Type>Literature</Reference_Type>  <Name>PubMed</Name>  <Description>Data curated from biomedical literature</Description>  <Record_ID>https://www.ncbi.nlm.nih.gov/pubmed/17505062 </Record_ID>  <Version>2007</Version>  <Acquisition_Date>2017</Acquisition_Date>  </Reference>  <Evidence_Score>  <Item>  <Key>Manual curation</Key>  <Score>1</Score>  </Item>  </Evidence_Score>  </CODA_Knowledge_Unit> |
| --- |

**Figure S5 Knowledge Unit (KU) Example 4.** ESR1 (ENSG00000091831) and BRCA1 (ENSG00000012048), two proteins physically interact with each other.

| <CODA_Knowledge_Unit>  <Subject>  <Entity>  <Entity_Core>  <Gene>  <Ref_Identifier>  <DBName>Ensembl</DBName>  <Identifier>ENSG00000091831</Identifier>  </Ref_Identifier>  </Gene>  </Entity_Core>  </Entity>  </Subject>  <Predicate>Undirected link</Predicate>  <Object>  <Entity>  <Entity_Core>  <Phenotype>  <Ref_Identifier>  <DBName>UMLS</DBName>  <Identifier>C0678222</Identifier>  </Ref_Identifier>  </Phenotype>  </Entity_Core>  </Entity>  </Object>  <Predicate_In_Reference>Marker/mechanism</Predicate_In_Reference>  <Species>  <Ref_Identifier>  <DBName>MeSH</DBName>  <Identifier>D006801</Identifier>  </Ref_Identifier>  </Species>  <Reference>  <Reference_Type>Database</Reference_Type>  <Name>CTD</Name>  <Description>Manually curated data</Description>  <Record_ID>ctdbase.org/detail.go?type=gene&amp;acc=2099&amp; view=disease</Record_ID>  <Version>2017</Version>  <Acquisition_Date>2017</Acquisition_Date>  </Reference>  <Evidence_Score>  <Item>  <Key>Manual curation</Key>  <Score>1</Score>  </Item>  </Evidence_Score>  </CODA_Knowledge_Unit> |
| --- |

**Figure S6 Knowledge Unit (KU) Example 5.** ESR1 (ENSG00000091831) is reported as marker or mechanism of breast cancer (C0678222).

| <CODA_Knowledge_Unit>  <Subject>  <Entity>  <Entity_Core>  <Gene>  <Ref_Identifier>  <DBName>Ensembl</DBName>  <Identifier>ENSG00000091831</Identifier>  </Ref_Identifier>  <Molecule_Specification>  <Molecule_Type>Protein</Molecule_Type>  </Molecule_Specification>  </Gene>  </Entity_Core>  </Entity>  </Subject>  <Predicate>Positive increase</Predicate>  <Object>  <Entity>  <Entity_Core>  <Gene>  <Ref_Identifier>  <DBName>Ensembl</DBName>  <Identifier>ENSG00000110092</Identifier>  </Ref_Identifier>  <Molecule_Specification>  <Molecule_Type>RNA</Molecule_Type>  </Molecule_Specification>  </Gene>  </Entity_Core>  </Entity>  </Object>  <Environmental_Context>  <Condition>  <Key>Phenotype</Key>  <Value>  <Ref_Identifier>  <DBName>UMLS</DBName>  <Identifier>C0678222</Identifier>  </Ref_Identifier>  </Value>  </Condition>  </Environmental_Context>  <Predicate_In_Reference>Expression</Predicate_In_Reference>  <Species>  <Ref_Identifier>  <DBName>MeSH</DBName>  <Identifier>D006801</Identifier>  </Ref_Identifier>  </Species>  <Reference>  <Reference_Type>Database</Reference_Type>  <Name>KEGG</Name>  <Description>Manually curated data</Description>  <Record_ID>www.genome.jp/kegg-bin/show_pathway?hsa05224 </Record_ID>  <Version>2017</Version>  <Acquisition_Date>2017</Acquisition_Date>  </Reference>  <Evidence_Score>  <Item>  <Key>Manual curation</Key>  <Score>1</Score>  </Item>  </Evidence_Score>  </CODA_Knowledge_Unit> |
| --- |

**Figure S7 Knowledge Unit (KU) Example 6.** In breast cancer pathway (C0678222), ESR1 (ENSG00000091831) increases expression level of CCND1 (ENSG00000110092).

| <CODA_Knowledge_Unit>  <Subject>  <Entity>  <Entity_Core>  <Compound>  <Ref_Identifier>  <DBName>STITCH</DBName>  <Identifier>CIDs00445154</Identifier>  </Ref_Identifier>  </Compound>  </Entity_Core>  </Entity>  </Subject>  <Predicate>Positive increase</Predicate>  <Object>  <Entity>  <Entity_Core>  <Compound>  <Ref_Identifier>  <DBName>STITCH</DBName>  <Identifier>CIDs00031703</Identifier>  </Ref_Identifier>  </Compound>  </Entity_Core>  </Entity>  </Object>  <Environmental_Context>  <Condition>  <Key>Cell line</Key>  <Value>  <Ref_Identifier>  <DBName>CLO</DBName>  <Identifier>CLO0007606</Identifier>  </Ref_Identifier>  </Value>  </Condition>  </Environmental_Context>  <Predicate_In_Reference>Associate</Predicate_In_Reference>  <Species>  <Ref_Identifier>  <DBName>MeSH</DBName>  <Identifier>D006801</Identifier>  </Ref_Identifier>  </Species>  <Reference>  <Reference_Type>Literature</Reference_Type>  <Name>PubMed</Name>  <Description>Data curated from biomedical literature</Description>  <Record_ID>https://www.ncbi.nlm.nih.gov/pubmed/24161697 </Record_ID>  <Version>2014</Version>  <Acquisition_Date>2017</Acquisition_Date>  </Reference>  <Evidence_Score>  <Item>  <Key>Manual curation</Key>  <Score>1</Score>  </Item>  </Evidence_Score>  </CODA_Knowledge_Unit> |
| --- |

**Figure S8 Knowledge Unit (KU) Example 7.** It was observed that resveratrol (CIDs00445154) increased cellular accumulation of doxorubicin (CIDs00031703) in combinatorial treatment in MCF-7 cell line (CLO0007606).

| <CODA_Knowledge_Unit>  <Subject>  <Entity>  <Entity_Core>  <Gene>  <Ref_Identifier>  <DBName>Ensembl</DBName>  <Identifier>ENSG00000110092</Identifier>  </Ref_Identifier>  <Molecule_Specification>  <Molecule_Type>Protein</Molecule_Type>  </Molecule_Specification>  </Gene>  <Gene>  <Ref_Identifier>  <DBName>Ensembl</DBName>  <Identifier>ENSG00000135446</Identifier>  </Ref_Identifier>  <Molecule_Specification>  <Molecule_Type>Protein</Molecule_Type>  </Molecule_Specification>  </Gene>  </Entity_Core>  </Entity>  </Subject>  <Predicate>Positive increase</Predicate>  <Object>  <Entity>  <Entity_Core>  <Gene>  <Ref_Identifier>  <DBName>Ensembl</DBName>  <Identifier>ENSG00000139687</Identifier>  </Ref_Identifier>  <Molecule_Specification>  <Molecule_Type>Protein</Molecule_Type>  <Molecule_Modification>Phosphorylation </Molecule_Modification>  </Molecule_Specification>  </Gene>  </Entity_Core>  </Entity>  </Object>  <Environmental_Context>  <Condition>  <Key>Phenotype</Key>  <Value>  <Ref_Identifier>  <DBName>UMLS</DBName>  <Identifier>C0678222</Identifier>  </Ref_Identifier>  </Value>  </Condition>  </Environmental_Context>  <Predicate_In_Reference>Phosphorylation</Predicate_In_Reference>  <Species>  <Ref_Identifier>  <DBName>MeSH</DBName>  <Identifier>D006801</Identifier>  </Ref_Identifier>  </Species>  <Reference>  <Reference_Type>Database</Reference_Type>  <Name>KEGG</Name>  <Description>Manually curated data</Description>  <Record_ID>www.genome.jp/kegg-bin/show_pathway?hsa05224 </Record_ID>  <Version>2017</Version>  <Acquisition_Date>2018</Acquisition_Date>  </Reference>  <Evidence_Score>  <Item>  <Key>Manual curation</Key>  <Score>1</Score>  </Item>  </Evidence_Score>  </CODA_Knowledge_Unit> |
| --- |

**Figure S9 Knowledge Unit (KU) Example 8.** A protein complex composed of CCND1 (ENSG00000110092) and CDK4 (ENSG00000135446) phosphorylates RB1 (ENSG00000139687) in a breast carcinoma condition (C0678222).

| <CODA_Knowledge_Unit>  <Subject>  <Entity>  <Entity_Core>  <Compound>  <Ref_Identifier>  <DBName>STITCH</DBName>  <Identifier>CIDs00001038</Identifier>  </Ref_Identifier>  </Compound>  </Entity_Core>  <Anatomical_Context>  <Organ>  <Ref_Identifier>  <DBName>MeSH</DBName>  <Identifier>D001921</Identifier>  </Ref_Identifier>  </Organ>  </Anatomical_Context>  </Entity>  <Entity>  <Entity_Core>  <Compound>  <Ref_Identifier>  <DBName>STITCH</DBName>  <Identifier>CIDs22833512</Identifier>  </Ref_Identifier>  </Compound>  </Entity_Core>  <Anatomical_Context>  <Organ>  <Ref_Identifier>  <DBName>MeSH</DBName>  <Identifier>D001921</Identifier>  </Ref_Identifier>  </Organ>  </Anatomical_Context>  </Entity>  <Entity>  <Entity_Core>  <Compound>  <Ref_Identifier>  <DBName>STITCH</DBName>  <Identifier>CIDs00005870</Identifier>  </Ref_Identifier>  </Compound>  </Entity_Core>  <Anatomical_Context>  <Organ>  <Ref_Identifier>  <DBName>MeSH</DBName>  <Identifier>D001921</Identifier>  </Ref_Identifier>  </Organ>  </Anatomical_Context>  </Entity>  <Entity>  <Entity_Core>  <Gene>  <Ref_Identifier>  <DBName>Ensembl</DBName>  <Identifier>ENSG00000108786</Identifier>  </Ref_Identifier>  <Molecule_Specification>  <Molecule_Type>Protein</Molecule_Type>  </Molecule_Specification>  </Gene>  </Entity_Core>  <Anatomical_Context>  <Organ>  <Ref_Identifier>  <DBName>MeSH</DBName>  <Identifier>D001921</Identifier>  </Ref_Identifier>  </Organ>  </Anatomical_Context>  </Entity>  </Subject>  <Predicate>Positive increase</Predicate>  <Object>  <Entity>  <Entity_Core>  <Compound>  <Ref_Identifier>  <DBName>STITCH</DBName>  <Identifier>CIDs00005886</Identifier>  </Ref_Identifier>  </Compound>  </Entity_Core>  <Anatomical_Context>  <Organ>  <Ref_Identifier>  <DBName>MeSH</DBName>  <Identifier>D001921</Identifier>  </Ref_Identifier>  </Organ>  </Anatomical_Context>  </Entity>  <Entity>  <Entity_Core>  <Compound>  <Ref_Identifier>  <DBName>STITCH</DBName>  <Identifier>CIDs00005757</Identifier>  </Ref_Identifier>  </Compound>  </Entity_Core>  <Anatomical_Context>  <Organ>  <Ref_Identifier>  <DBName>MeSH</DBName>  <Identifier>D001921</Identifier>  </Ref_Identifier>  </Organ>  </Anatomical_Context>  </Entity>  </Object>  <Predicate_In_Reference>React</Predicate_In_Reference>  <Species>  <Ref_Identifier>  <DBName>MeSH</DBName>  <Identifier>D006801</Identifier>  </Ref_Identifier>  </Species>  <Reference>  <Reference_Type>Database</Reference_Type>  <Name>Recon2</Name>  <Description>Manually curated data</Description>  <Record_ID>https://vmh.uni.lu/#reaction/HSD17B1</Record_ID>  <Version>2017</Version>  <Acquisition_Date>2017</Acquisition_Date>  </Reference>  <Evidence_Score>  <Item>  <Key>Manual curation</Key>  <Score>1</Score>  </Item>  </Evidence_Score>  </CODA_Knowledge_Unit> |
| --- |

**Figure S10 Knowledge Unit (KU) Example 9.** A metabolic reaction in which estrone (CIDs00005870) is converted into estradiol (CIDs00005757), catalyzed by HSD17B1 (ENSG00000108786) in the human brain (D001921).
